# Application of annotation-agnostic RNA sequencing data analysis tools for biomarker discovery in liquid biopsy

**DOI:** 10.3389/fbinf.2023.1127661

**Published:** 2023-04-28

**Authors:** Gabriel Wajnberg, Eric P. Allain, Jeremy W. Roy, Shruti Srivastava, Daniel Saucier, Pier Morin, Alier Marrero, Colleen O’Connell, Anirban Ghosh, Stephen M. Lewis, Rodney J. Ouellette, Nicolas Crapoulet

**Affiliations:** ^1^ Atlantic Cancer Research Institute, Moncton, NB, Canada; ^2^ Department of Clinical Genetics, Vitalité Health Network, Dr. Georges-L.-Dumont University Hospital Centre, Moncton, NB, Canada; ^3^ Department of Chemistry and Biochemistry, Université de Moncton, Moncton, NB, Canada; ^4^ Beatrice Hunter Cancer Research Institute, Halifax, NS, Canada; ^5^ Dr. Georges-L.-Dumont University Hospital Centre, Moncton, NB, Canada; ^6^ Stan Cassidy Centre for Rehabilitation, Fredericton, NB, Canada

**Keywords:** small RNA, extracellular vesicles, annotation-agnostic, quantification algorithms, biomarkers, liquid biopsy, genetic diseases

## Abstract

RNA sequencing analysis is an important field in the study of extracellular vesicles (EVs), as these particles contain a variety of RNA species that may have diagnostic, prognostic and predictive value. Many of the bioinformatics tools currently used to analyze EV cargo rely on third-party annotations. Recently, analysis of unannotated expressed RNAs has become of interest, since these may provide complementary information to traditional annotated biomarkers or may help refine biological signatures used in machine learning by including unknown regions. Here we perform a comparative analysis of annotation-free and classical read-summarization tools for the analysis of RNA sequencing data generated for EVs isolated from persons with amyotrophic lateral sclerosis (ALS) and healthy donors. Differential expression analysis and digital-droplet PCR validation of unannotated RNAs also confirmed their existence and demonstrates the usefulness of including such potential biomarkers in transcriptome analysis. We show that find-then-annotate methods perform similarly to standard tools for the analysis of known features, and can also identify unannotated expressed RNAs, two of which were validated as overexpressed in ALS samples. We demonstrate that these tools can therefore be used for a stand-alone analysis or easily integrated into current workflows and may be useful for re-analysis as annotations can be integrated *post hoc*.

## Introduction

Liquid biopsy is a broad term used to describe the collection of biological fluids, such as blood, urine, and saliva, to identify biomarkers associated with a specific disease (Mader and Pantel, 2017). This approach can be beneficial for sampling diseased tissues that would otherwise be inaccessible or require invasive sampling methods. The biological fluids sampled by liquid biopsy approaches contain circulating tumor cells (CTCs), circulating cell-free DNA (cfDNA), circulating cell-free RNA (cfRNA), proteins, metabolites and extracellular vesicles (EVs), all of which contain potential biomarkers (Heitzer et al., 2019). In recent years, liquid biopsies have been studied in the context of personalized medicine due to their promise as a tool for diagnostics, monitoring disease, and prognostication (Mader and Pantel, 2017); however, as this field evolves so do the technological challenges associated with sequencing analysis of liquid biopsies. One such challenge in this field is the processing and analysis of transcriptomic data generated from liquid biopsy samples. We have therefore chosen to focus on improving the analytical methods applied to data generated from RNA sequencing analyses of EVs.

EVs can be categorized into two different classes: exosomes and ectosomes (Thery et al., 2018; van Niel et al., 2018). These small structures are released from different cell types and contain various biomolecules such as nucleic acids, proteins, and metabolites (Vagner et al., 2018; Pathan et al., 2019). Among the nucleic acids contained within EVs, there is a diversity of RNA types, including messenger RNA (mRNA), microRNA (miRNA), long non-coding RNA (lncRNA), ribosomal RNA (rRNA), piwi-RNA (piRNA), circular RNA (circRNA), transfer RNA (tRNA), small nuclear RNA (snRNA), and small nucleolar RNA (snoRNA) (Perez-Boza et al., 2018; Liu et al., 2019; Turchinovich et al., 2019); however, most RNA sequencing (RNA-Seq) studies in the EV field focus on the analysis of miRNA and lncRNA. As a result, this field has relied on methods and tools developed for the analysis of both small RNA sequencing (sRNA-Seq) and standard RNA-Seq. The expression patterns of RNA determined for liquid biopsies can be indicative of disease onset or progression and can therefore represent an important tool for patient monitoring. Therefore, it is essential to analyze RNA-Seq data obtained from liquid biopsies in a way that yields the most potential biomarkers accurately.

Many current RNA sequencing analysis pipelines utilize read summarization software, such as Stringtie, HTSeq, and featureCounts, to obtain read counts for known features (Liao et al., 2014; Anders et al., 2015; Pertea et al., 2015). These algorithms are highly useful but present some critical limitations. For example, these tools require an annotation file that contains the chromosome position of genes, transcripts, or exons, which may be subject to frequent updates over time as annotations become complete or change in structure. Consequently, it may require frequent re-analysis of data using the most up-to-date annotations to detect newly discovered features. In general, these algorithms can only call one RNA feature at a time (the user must modify the annotation file to identify multiple RNA types simultaneously) and they are not designed to find unannotated expressed regions. Recently, the Extracellular RNA Communication Consortium (ERCC) recommended a new pipeline, exceRpt, which can deal with several RNA types; however, this pipeline also relies on known features, as it performs multiple stepwise assignments of reads to prioritized annotations in a hierarchical fashion (Rozowsky et al., 2019). Presently, this pipeline is the gold-standard bioinformatics pipeline for analyzing small RNA sequencing data from EVs. Other useful pipelines and tools such as sRNAbench, Oasis 2, sRNAPipe, miRDeep2, sRNAtoolbox, and sRNAnalyzer are well-adapted for small RNA and are often used for analyzing sequence data from EV samples or integrated into larger pipelines (Friedlander et al., 2008; Friedlander et al., 2012; Rueda et al., 2015; Wu et al., 2017; Pogorelcnik et al., 2018; Rahman et al., 2018; Paricio-Puerta et al., 2019). Some, such as miRDeep2, Oasis 2, and sRNAbench can be used for *de novo* RNA discovery; however, these tools frequently require some form of annotation for differential expression analysis. Most of these tools apply some form of successive alignment steps to various annotations, similar to exceRpt. Alignment-free methods have recently gained significant popularity in genomics and have been adapted to sRNA-Seq with the development of DEUS, an R package for small RNA profiling that is based on the Differential Expression of Unique Sequences (Jeske et al., 2019).

An alternative approach to the above-described pipelines is to acquire the read counts per expressed region and subsequently annotate the reads *post hoc*. This approach is of particular interest in the context of sequencing EV nucleic acid cargo, which consists of diverse RNA types in relatively low quantities. We chose to explore this approach by using three annotation-agnostic tools: derfinder, ShortStack and srnadiff (Axtell, 2013; Collado-Torres et al., 2017; Zytnicki and Gonzalez, 2021). While not yet extensively used in the liquid biopsy field, these tools offer certain advantages when compared to feature-based software. We assessed the flexibility of find-then-annotate methods for the analysis of multiple RNA types using known annotations. We also compared count summarization and differential expression results for annotated features among standard tools and region-based tools, which were annotated after quantification. Lastly, we validated potential unannotated diagnostic RNA biomarkers in EVs sampled from a group of persons with ALS using annotation-agnostic approaches with total RNA, as this strategy is rapidly becoming of interest in the liquid biopsy field (von Felden et al., 2021).

Our results show that find-then-annotate approaches can be successfully applied for the identification of multiple RNA types using sequencing data obtained from EVs. We demonstrate that annotation-agnostic tools yield similar results to other standard methods for known features while identifying *de novo* additional expressed regions packaged into the EVs of persons with ALS. This approach expands the pool of possible biomarkers in liquid biopsy experiments by considering expressed regions individually rather than as part of a larger feature, and by including orphan expressed regions of possible biological significance. Importantly, we are able to achieve these results in a few steps, using readily available software.

## Methods

### Subjects and samples

Previously generated RNA sequencing data for EVs isolated from 14 plasma samples (eight ALS and six healthy donors) was used for these analyses (Saucier et al., 2019). Plasma samples were obtained from donors who had given informed consent in accordance with study protocol, as accepted by Vitalité and Horizon Health Networks Ethics Boards (New Brunswick, Canada). EV isolation, RNA-seq library preparation, and sequencing experiments were carried out as previously described (Saucier et al., 2019). The data used in this study is a sub-set of the donors analyzed by Saucier et al. (2019). Public data for comparing tools was accessed through the Sequence Read Archive (SRA) and Gene Expression Omnibus (GEO) with accession number GSE67004 (Cha et al., 2015).

### Read summarization and differential expression analysis

We performed the alignment of our small RNA seq data (Saucier et al., 2019) with bowtie2 (version 2.3.4.3) (Langmead et al., 2009) to the human reference genome GRCh37/hg19 using the following parameters: –local –very-sensitive-local –mm –q, and subsequently used samtools (version 1.6) for sorting and indexing (Li et al., 2009). The sorted bam files were used as input for derfinder (version 1.18.9) (Collado-Torres et al., 2017) and srnadiff (version 1.8.0) (Zytnicki and Gonzalez, 2021) for R (version 3.6.1) (R Core Team, 2021). After obtaining the expression counts matrices, we annotated each expressed region to the gencode annotation (version 19). For comparisons with featureCounts and HTSeq, reads in all expressed regions that overlapped a known gene were summed and counted towards that gene. Read summarization was performed using featureCounts with parameters: -T 8 -t “gene” --largestOverlap --ignoreDup --minOverlap 5 -C -M -O–o; and HTSeq with parameters: -f bam -a 0 -q -r pos -s no -t gene --idattr gene_id --nonunique all. ShortStack was run with default parameters and option --mincov 1. Differential expression analysis was performed with edgeR (version 3.26.8) (Vienna et al., 2010). The code used in these experiments is available at https://github.com/acri-nb/derfinder-pipe.

### Simulation

A 12-sample small RNA-seq experiment was simulated using the polyester package for R (version 1.20.0) (Frazee et al., 2015). Reads were 22 nucleotides in length with two 6-sample experimental groups. The ‘treatment’ condition had 20% of features with at least a two-fold increase over control, while 20% had a two-fold decrease. Only transcripts from chromosome 13 were generated to alleviate the computational load. Simulated reads were subjected to differential gene expression analysis using derfinder or ShortStack (annotation-agnostic tools) or using two popular annotation-based methods: HTSeq and featureCounts. Count matrices for specific regions detected with derfinder were generated using the regionMatrix function. Count matrices for ShortStack were produced by the command-line tool. Regions in these matrices were then assigned to known features using the GenomicRanges package and compared to results from featureCounts and HTSeq. To obtain count matrices from srnadiff results, srnadiff was run in ‘annotation’ mode prior to extracting count matrices.

### Digital droplet PCR validation of unannotated RNA sequences

Amplification and validation of unannotated RNA sequences were performed similarly to other small RNA (miRNA) as previously described (Saucier et al., 2019) using reverse transcription digital droplet PCR. Forward primer sequences used were 5ʹ- TCCTGTACTGAGTGCCC - 3ʹ for target 1, and 5ʹ -CTG​AGG​GGG​CAG​AGA​GCG​AGA​CT - 3ʹ for target 2. Reverse transcription efficiency was assessed using the internal miRTC control as described in the miScript Kit (Qiagen, Toronto, ON, Canada). The copies/μL for both targets were normalized to the copies/μL for miRTC.

### Data availability

All relevant sequencing data used in these experiments were deposited to the Gene Expression Omnibus (GEO) with the accession number GSE183942.

## Results

### The performance of annotation-agnostic software is similar to read-summarization in annotated genomic regions with simulated data

**FIGURE 1:**
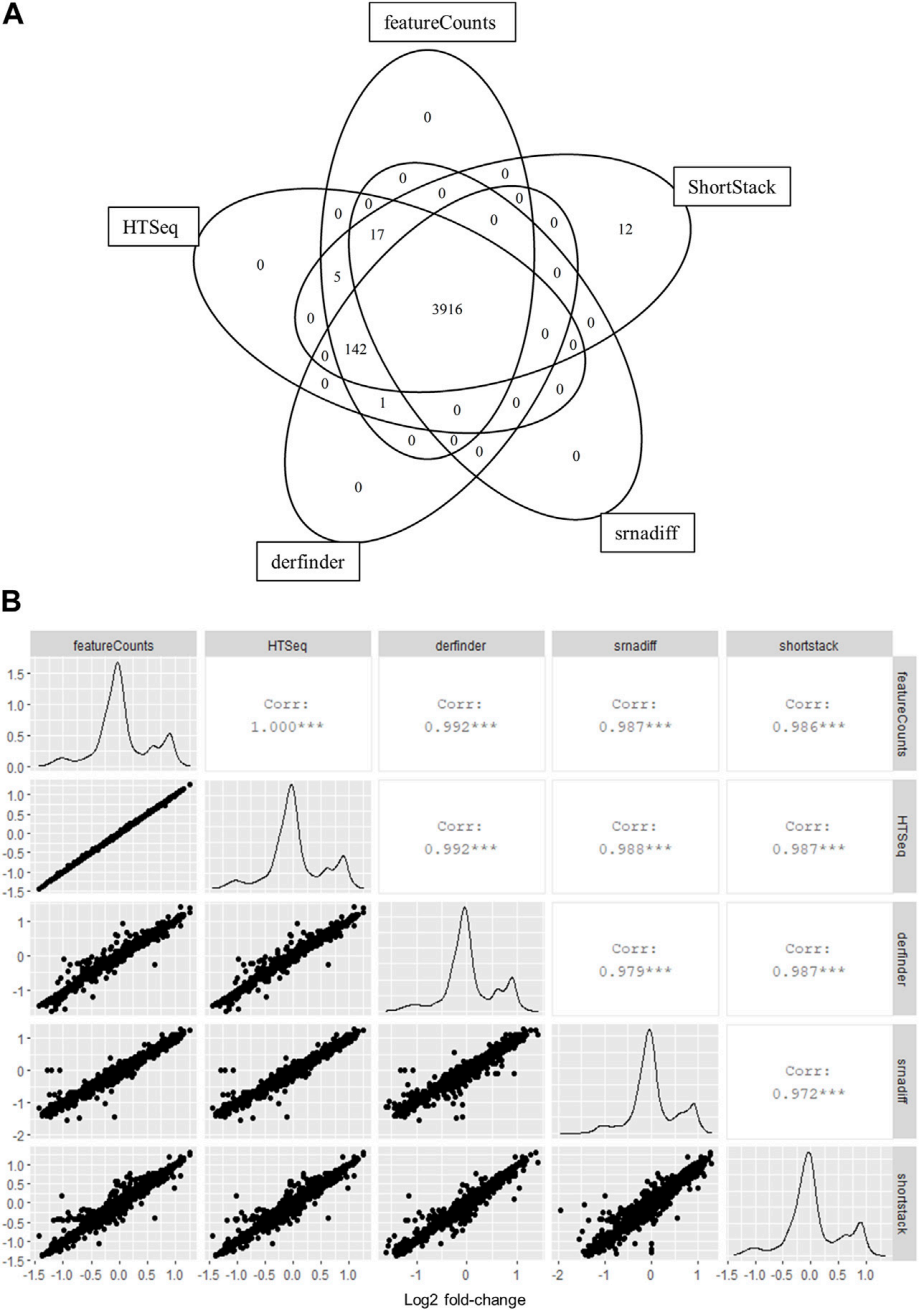
Annotation-agnostic tools yield similar results to other read summarization software for simulated RNA-seq experiments. **(A)** Number of features detected by each tool after low-expression filtering. A 12-sample small RNA-seq experiment was simulated using the polyester package for R. Two six-sample groups were assigned, with 20% of features showing a 2-fold upregulation, while another 20% were downregulated by 2-fold in the ‘treatment’ group. Reads were counted on overlapping features with derfinder and ShortStack, while srnadiff was run in “annotation” mode for comparisons with featureCounts and HTSeq. **(B)** Log2 fold-change values per gene were calculated between groups using edgeR and compared for each tool.

We first chose to test the applicability of annotation-agnostic tools for RNA sequencing (RNA-seq) data using a simulated dataset. RNA-seq reads were simulated using the polyester package for R before analysis using derfinder, srnadiff, ShortStack or two popular annotation-based quantification methods: HTSeq and featureCounts. Count matrices for specific regions detected with derfinder were generated using the regionMatrix function. Regions in this matrix were then assigned to known features using the GenomicRanges package. Results from derfinder, ShortStack and srnadiff were then compared to those from featureCounts and HTSeq. Feature-wise counts generated by annotation-agnostic methods were highly correlated to those from featureCounts and those from HTSeq for all 12 simulated samples (Figure 1A; Supplementary Table S1). Out of 4,093 features quantified by at least one tool, 96% (3,916) had also been detected by all other tools (Figure 1A). Only 12 features were solely detected by only one method. Furthermore, differential expression analysis of simulated data showed similar effect sizes, sensitivity and specificity among tools, assuming a complete annotation (Figure 1B; Supplementary Table S2). Furthermore, we ran an identical analysis on real EV data of KRAS mutant and wild-type cell lines from Cha et al. (2015) downloaded from SRA to confirm performance comparisons in a second dataset. Differential expression analysis between EVs from KRAS-mutant DKO-1 cells and those from wild-type DKs-8 cells was used as the ground truth by using the authors’ quantifications, supplied as raw miRNA count matrices on GEO. Afterward, differential expression was carried out using count matrices generated by all other tools and then compared to the authors’ results. Sensitivity was between 0.73 and 0.8 for annotation-based tools and between 0.67 and 0.69 in annotation agnostic methods. Specificity was approximately 0.95 for all tools (Supplementary Table S3). Calculated effect sizes were highly correlated to the truth set (Supplementary Figure S1).

### Feature quantification of RNA-seq data obtained from the EVs of persons with ALS using annotation-agnostic software is similar to standard methods

**FIGURE 2:**
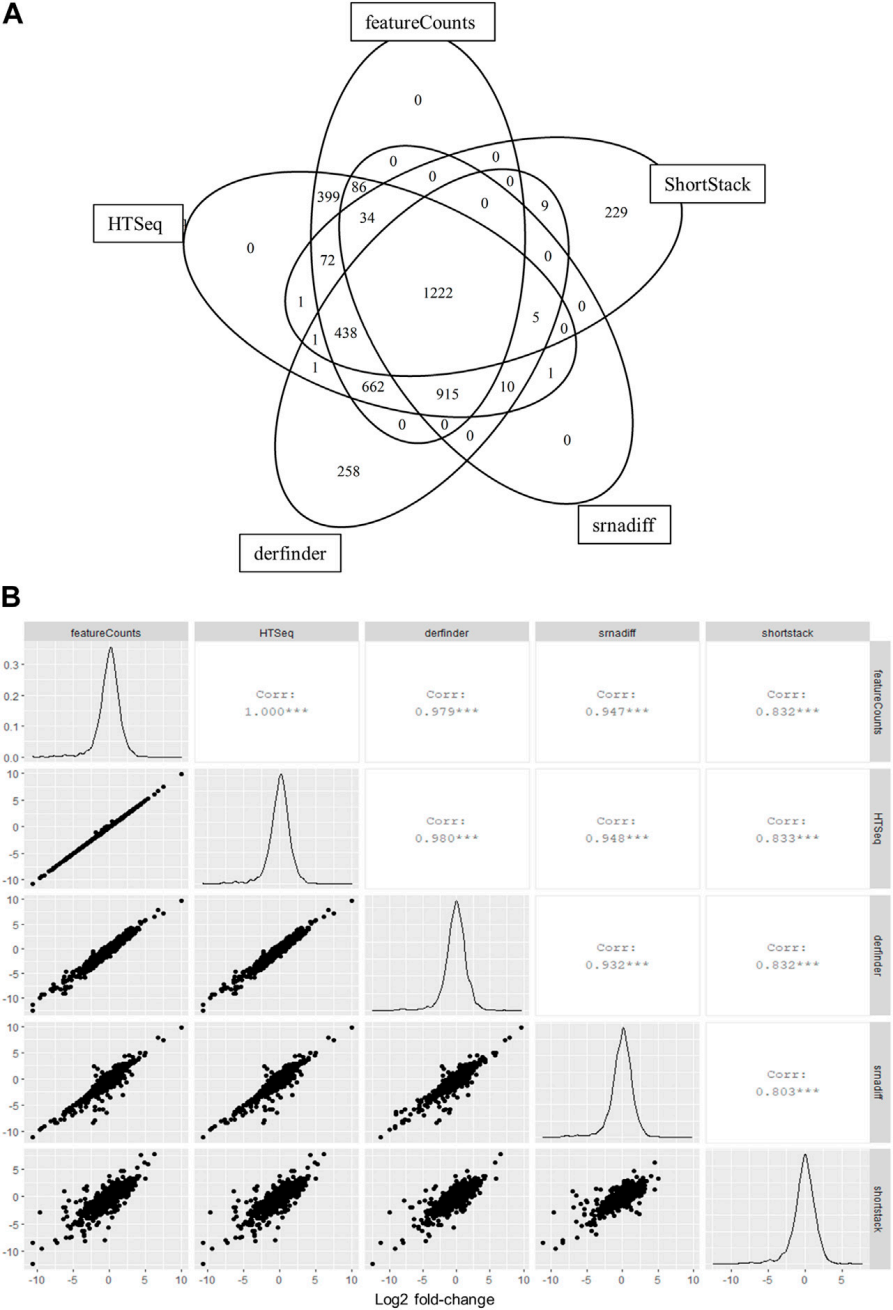
Annotation-agnostic tools yield similar results to other read summarization software with real RNA-seq data. **(A)** Number of features detected by each tool after low-expression filtering. EVs were isolated from blood samples of 8 persons with ALS and 6 healthy controls and subjected to RNA-sequencing, as previously described (Saucier et al., 2019). **(B)** Log2 fold-change values calculated among groups using edgeR and compared for each bioinformatic tool.

We were interested in testing the applicability of annotation-agnostic tools for the analysis of small RNA-seq data obtained from liquid biopsy material, namely, EVs. We therefore sought to compare results from annotation-free methods to those from other standard tools that summarize reads to feature-level using real RNA-seq data from EVs, which was previously reported in Saucier et al. (2019). Gene-level counts calculated using derfinder were highly correlated to counts obtained from annotation-based methods, while srnadiff and ShortStack were moderately correlated (Supplementary Table S4). A total of 4,343 features were identified, with 3,856 features (89%) detected by at least two tools and 1,222 features (28%) detected by all tools (Figure 2A). Gene-wise effect sizes (log2 fold changes) between ALS samples and healthy donors were all highly correlated among tools (Figure 2B).

**FIGURE 3:**
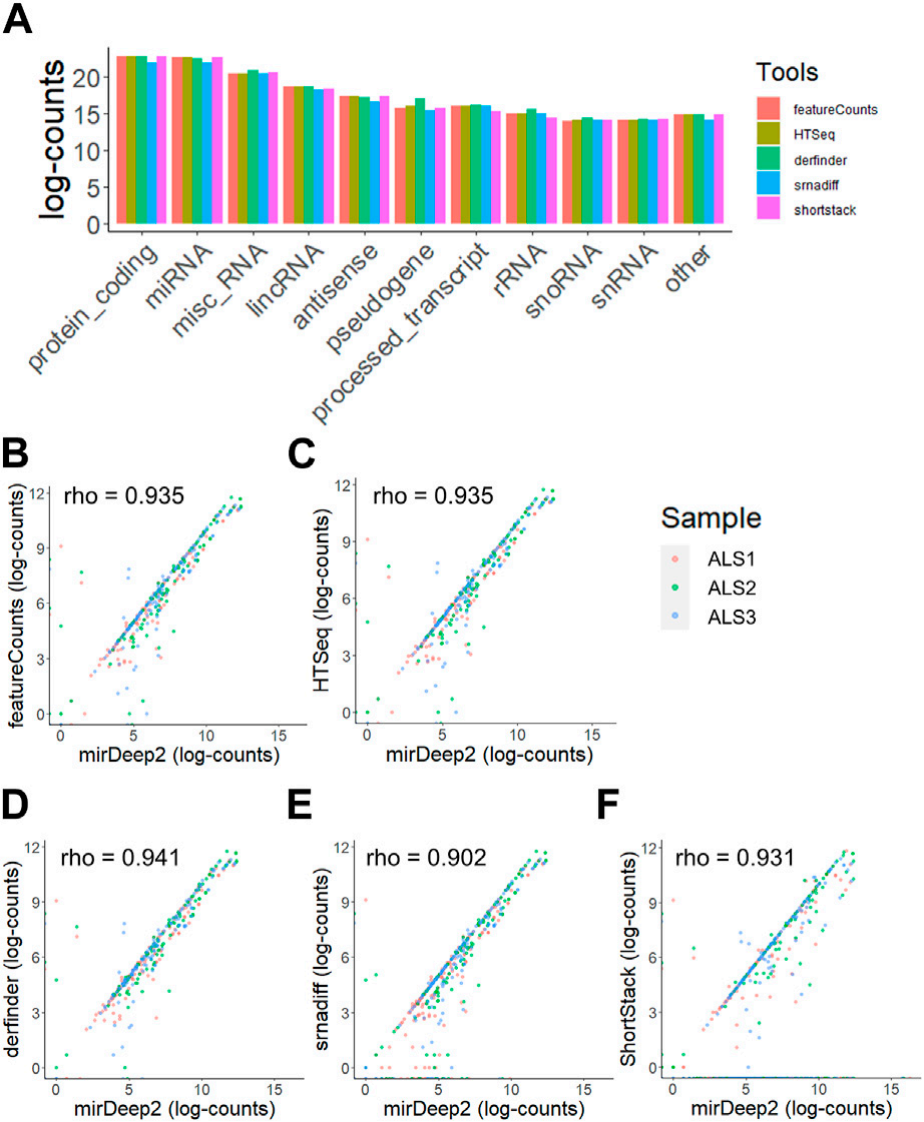
Distribution of reads across RNA types is not altered when using agnostic tools. **(A)** The distribution of reads aligning to specific subclasses of RNA when using annotation-free methods. **(B–F)** Comparative quantification and rank correlation of miRNA with miRDeep2, featureCounts **(B)**, HTSeq **(C)**, derfinder **(D)**, srnadiff **(E)**, and ShortStack **(F)** in three samples.

Furthermore, when quantifying total RNA, the relative abundances of different RNA species such as mRNA, miRNA and lncRNA did not differ substantially between both strategies, with most reads aligning to miRNA, mRNA or lncRNA, which is typical of extracellular RNA sequencing data (Liu et al., 2019) (Figure 3A). miRNA quantification results from derfinder, ShortStack and srnadiff were also compared to mirDeep2, which is explicitly designed for miRNA analysis. Raw miRNA counts from mirDeep2 were compared in a pairwise fashion to each of the other tools (featureCounts, HTSeq, derfinder, ShortStack and srnadiff) for three random samples and showed a high concordance for all tools (Figures 3B–F).

Another frequently used method for analyzing sequencing data in EV genomics consists of aligning reads directly to known transcript annotations, such as miRbase. This was the original approach used for miRNA analysis of the published sample data (Saucier et al., 2019). We compared miRNA counts from samples mapped directly to miRbase annotations or quantified using derfinder, ShortStack srnadiff, featureCounts and HTSeq. Similar results to miRbase mapping were achieved using all five methods when considering sample-wise normalized counts, log2 fold-change and false discovery rate (FDR), as calculated using the edgeR package (Supplementary Table S5). Despite small differences in the total number of miRNAs identified among tools, comparisons were done using miRNAs common to all analyses. In sum, these results suggest that annotation-agnostic approaches may be suitable for the analysis of small RNA-Seq data and are concordant with tools designed for traditional RNA-seq data.

### Standardized differential gene expression analysis leads to similar biological conclusions when using annotation-agnostic tools for feature-level analysis

We then sought to compare differential gene expression analysis among all tools to determine if the number of statistically significant results varied among methods. Differential gene expression analysis between persons with ALS and healthy donors was performed using a standardized analysis with the edgeR package. As subtle differences were evident in feature quantification and stringency among tools, optimal low-count filter and alpha thresholds were determined iteratively for each tool by comparing differential expression results to those from featureCounts. This allowed us to determine the parameters that yield the results most similar to featureCounts. Using these thresholds, there were 155 and 153 significantly differentially expressed genes identified by HTSeq and featureCounts, respectively. Using this same threshold, we observed 137, 84, and 121 significantly differentially expressed genes using derfinder, ShortStack, and srnadiff, respectively. All four tools identified 24 (10% of all significant results) common features as significantly differentially expressed, while 163 (68% of all significant results) features were detected by two or more tools (Supplementary Figure S2). Afterward, hypergeometric tests of pathway and gene ontology (GO) enrichment were performed with statistically significant results from each tool. The resulting term lists were sorted by adjusted *p*-value and rank-correlated. This analysis showed a good (>0.6) correlation of gene ontology (GO) enrichment terms among all tools (Supplementary Table S6). The same trend was observed with pathway enrichment results from KEGG; however, the correlations between annotation-free methods and classical tools for pathways identified were weaker (Supplementary Table S7). Gene-set enrichment analysis (GSEA) using GO terms and the KEGG pathway database resulted in similar trends (not shown).

### Annotation-agnostic tools detect orphan expressed regions in EV sequence data that may represent novel biomarkers

We then generated an R script that outputs expressed regions detected by derfinder and Shortstack with several supplementary columns describing overlaps with known features in the provided annotation. When run agnostically and considering only expressed regions rather than genes, 2,672, 2,737, and 2,473 expressed regions were identified by derfinder, ShortStack, and srnadiff after low-count filtering, respectively. Regions identified by all three tools were chosen for subsequent validation by qPCR. For this analysis, alpha was set to 0.1 to maximize the number of candidate biomarker regions.

**FIGURE 4:**
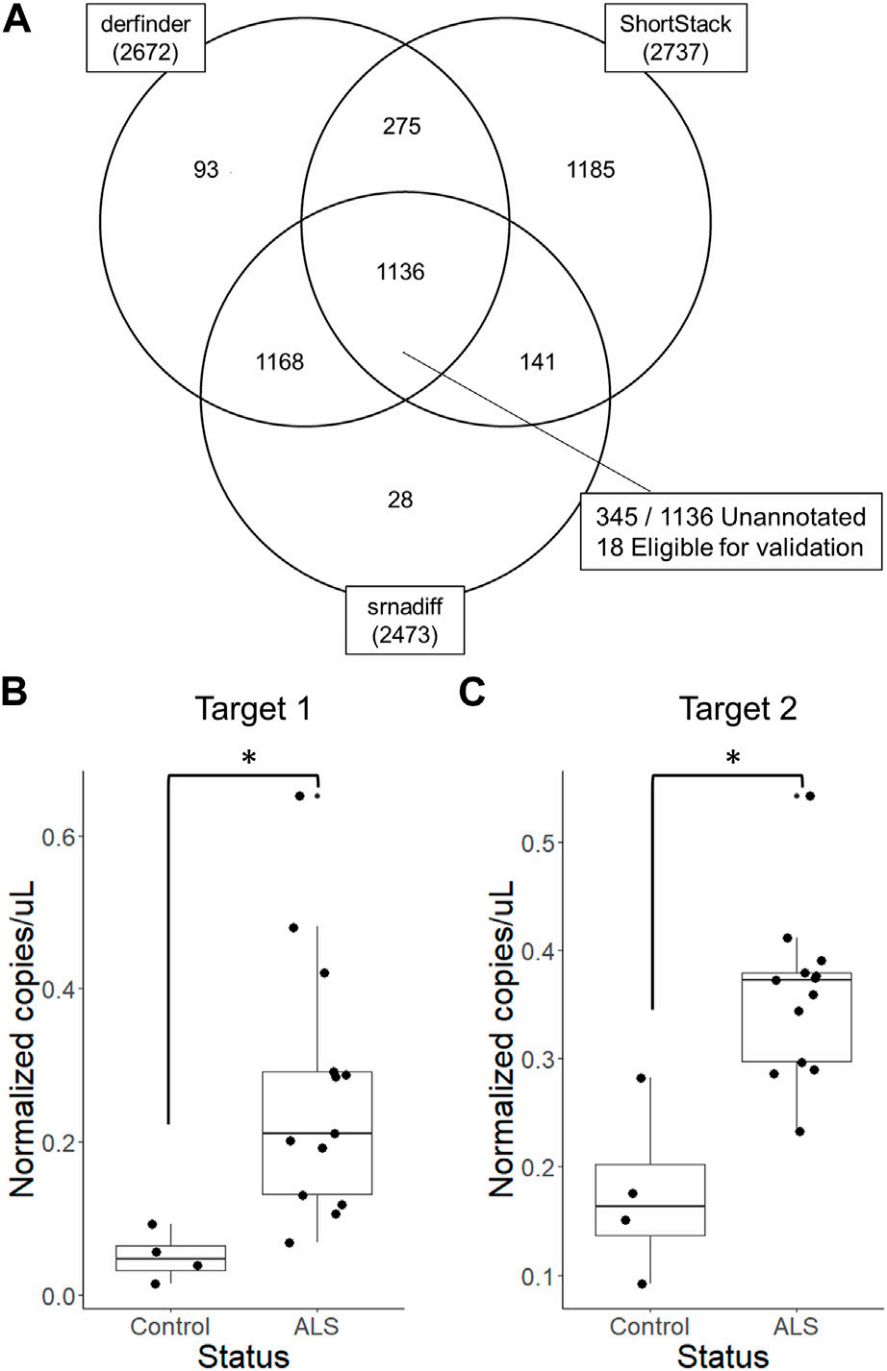
Validation of differentially expressed unannotated regions by reverse transcription digital droplet PCR **(A)** Two unannotated regions were chosen based on derfinder, ShortStack, and srnadiff results and validated using reverse transcription digital droplet PCR. Targets were significantly differentially expressed unannotated regions identified by derfinder, ShortStack and srnadiff, that do not align to exogenous genomes or known features. **(B)** The first target region was located at chr1:186673706–186673729 (hg19) and **(C)** the second was located at chr8:125980908–125980931 (hg19). *p < 0.05, Wilcoxon rank-sum exact test.

Unannotated expressed regions were mapped to human and exogenous genomes to omit possible confounding sequences, as small RNAs often also map to exogenous organisms. Using BLAST, 56% of unannotated expressed regions could also be mapped to exogenous genomes (not shown). These regions were excluded from downstream validation. Following these analyses, three targets were chosen for subsequent validation by reverse transcription digital droplet PCR, two of which could be successfully amplified. Both RNAs were highly abundant, more than 20 nucleotides in length, and significantly differentially expressed in ALS patient EVs compared to EVs from healthy donors with all three tools (Figure 4, Supplementary Figures S3, S4; Supplementary Table S8). Fifteen other unannotated regions fit these criteria but were not investigated further. RNAs chosen for validation also did not map to any recently discovered genomic features, as determined by BLAST.

## Discussion

The use of annotation-based approaches, such as direct alignment to an annotation database or application of read-summarization software, for RNA-sequencing data analysis, has many advantages. These approaches quantify read counts within the scope of known high-quality annotations, which increases the interpretability of the data generated by RNA-sequencing experiments. It may also help in avoiding the interpretation of repetitive or otherwise problematic loci; however, some information is lost when restricting analyses to known regions. Post-transcriptional processing of RNA species and incomplete annotations may lead to expressed regions that may not be identified with annotation-based approaches. Reads mapping to regions outside of known annotations are perhaps not as interpretable as those aligning to genomic features; however, they could be valuable in *de novo* biomarker discovery and machine learning, especially for liquid biopsies. Thus, in some cases, it may be worthwhile to include expressed RNAs from loci usually considered uninformative, especially with supervised learning algorithms, where the end goal may not necessarily be maximizing interpretability. The field of liquid biopsy research shares many of the challenges of sRNA-Seq analyses since small RNA species are often the most abundantly reported class of RNAs packaged into EVs. In addition, the majority of mRNA and lncRNA sequences detected in EVs are present as fragments of these large RNA species. These challenges are further complicated for EV analyses due to the complex and variable nature of sequencing data for EVs that is caused by multiple cell types contributing to the overall circulating EV pool in most biofluids. It is therefore of prime importance that the bioinformatic methods available for these types of analyses be as accurate, accessible, and complete as possible. Several tools designed specifically for miRNA analysis have been developed (Mathelier and Carbone, 2010; Friedlander et al., 2012; An et al., 2013; Lei and Sun, 2014; Higashi et al., 2015), but few tools exist for quantifying multiple RNA subtypes together or total RNA without annotations. Tools such as derfinder, ShortStack, and srnadiff have a broad scope beyond miRNA and mRNA, and could therefore be useful for the integration of many RNA types in expression analysis pipelines (Axtell, 2013; Stocks et al., 2018). Herein, we show that annotation-agnostic tools are highly flexible, as they generate results that compare to annotation-based tools (including featureCounts and HTSeq) when used in a standard differential expression context, however, these methods have the advantage of detecting unannotated regions. As shown in our simulation experiment, this allows the user to choose the level of either breadth or interpretability of the results, depending on planned downstream analyses.

Using biological data from Saucier et al. (2019) we have further demonstrated the extent of overlap between annotation-based and annotation-agnostic software. Most quantified features or regions are detected by all methods, with substantial overlap when using at least two tools. Count matrices generated by derfinder, ShortStack, HTSeq, and featureCounts often have a noticeably larger number of detected features when compared to srnadiff, likely due to differential management of overlapping genomic features and reads mapping to multiple regions of the reference genome; however, the count matrix returned by srnadiff largely correlates with other tools, both in terms of intersecting feature labels and read count. Consequently, effect sizes and statistics are similar for all five tools. Variability in results among tools could often be explained by irregular feature coverage, management of multi-mapping, or effects driven by a single sample.

Gene-ontology analysis of results from each tool indicates good *p*-value rank correlation among approaches, indicating that enriched GO terms are likely consistent, regardless of the choice of method. Pathway analysis is fairly correlated among derfinder, HTSeq, featureCounts, and srnadiff; however, pathway enrichment results from ShortStack do not correlate with those from other tools. This indicates that the conclusions drawn from results generated by annotation-agnostic methods generally resemble those from other approaches if using GO annotations, but may show substantial differences depending on which database is queried, especially for pathway enrichment databases. We also observed that srnadiff identifies more significantly differentially expressed regions than other tools when using built-in statistical methods. Therefore, we suggest that anyone considering the analysis of EV data with annotation-agnostic approaches make use of the output of multiple tools, and take great care in the choice of alignment and quantification parameters.

Here we assigned annotations using a single annotation file from GENCODE, which contains protein coding, miRNAs, lncRNAs, tRNAs, and other non-coding RNAs; however, it is possible to provide a custom annotation file as input for the method, such as piRNAs at pirBase (Wang et al., 2019) or tRNA-derived fragments in the MINT database (Pliatsika et al., 2018). Such custom annotation provides agnostic approaches with the flexibility to analyze sequencing data from total RNA when users have annotations that include many different RNA types. Consolidation of annotations, therefore, simplifies this type of analysis. Generally, software that makes use of reference genomes to identify *de novo* expressed regions is advantageous for small RNA, as these RNAs tend to be challenging to annotate due to ambiguity in feature start and end coordinates (Mohorianu et al., 2013). Nevertheless, as the tools available for identifying small RNA loci and the small RNA annotations themselves increase in quality, future data analyses and re-analysis of old data will also improve. We also purposefully chose an older GENCODE version, as this allowed us to verify if any detected regions we identified as unannotated are found to correspond to miRNAs in later versions of miRbase or GENCODE.

Validation of our data confirmed two unannotated regions as potential biomarkers for ALS, as these were highly over-expressed in ALS samples compared to healthy donors. Caution must be taken when choosing candidate targets for validation, as these may align to recent annotation releases and also to exogenous species, especially for short sequences. Bacterial RNA may be detected in circulation, and while we have verified this on an *ad hoc* basis for the targets chosen in the context of this comparative study, this should be done systematically, as is currently implemented in the exeRpt pipeline (Whittle et al., 2018; Rozowsky et al., 2019). In addition, while our analysis is mainly intended to provide evidence of usability for annotation-free tools in the field of EV research, we do recommend users to reflect on how to manage the idiosyncrasies of various small RNA types prior to use with real data. In sum, we show here that annotation-agnostic approaches to RNA-seq analysis are appropriate for analyzing mRNA, miRNA, or other small RNA in EVs and can also yield results that are generally comparable to other widely-used software such as HTSeq and featureCounts. We believe that this annotation-agnostic approach is well-adapted to analyze small RNAs and with additional interpretation may be conveniently adapted to analyze total RNA through the combination of derfinder, ShortStack, and srnadiff, and thus may improve the accuracy of biomarker discovery.

## Data Availability

Publicly available datasets were analyzed in this study. This data can be found here: https://www.ncbi.nlm.nih.gov/geo/query/acc.cgi?acc=GSE183942.
