# Supplementary material for: Application of annotation-agnostic RNA sequencing data analysis tools for biomarker discovery in liquid biopsy
Source: Front Bioinform. 2023 Apr 28;3:1127661. doi: 10.3389/fbinf.2023.1127661 (PMC10213969; doi:10.3389/fbinf.2023.1127661)
Supplement: Supplementary file 2 [file DataSheet1.PDF]

Table S1. Spearman correlation of feature quantification between annotation-based tools and annotation-agnostic methods with simulated data.

Tool Type	Tool Name	HTSeq	derfinder	srnadiff	ShortStack
Annotation-based	featureCounts	**1.000 ± 0.000**	**0.988 ± 0.001**	**0.884 ± 0.002**	**0.956 ± 0.012**
	HTSeq		**0.989 ± 0.001**	**0.884 ± 0.002**	**0.957 ± 0.012**
Annotation-agnostic	derfinder			**0.874 ± 0.003**	**0.965 ± 0.012**
	srnadiff				**0.843 ± 0.011**
	ShortStack				

Known features were quantified using featureCounts, HTSeq, derfinder, srnadiff, and ShortStack. Read counts for simulated features on chromosome 13 were correlated between methods on a per-sample basis. Values represent mean Spearman's rho ± standard deviation. Statistically significant correlations ($P<0.05$) are shown in bold.

Table S2. Sensitivity and specificity of differential gene expression analysis in simulated data for derfinder, ShortStack, srnadiff, featureCounts and HTSeq.

Tool Type	Tool	Sensitivity	Specificity
Annotation-based	featureCounts	0.61	0.94
	HTSeq	0.61	0.94
Annotation-agnostic	derfinder	0.60	0.93
	snradiff	0.62	0.96
	ShortStack	0.60	0.93

Standardized differential expression analysis was performed with the edgeR package using simulated data. Sensitivity and specificity were calculated based on the true positives and true negatives table given by the polyester R package.

Table S3. Sensitivity and specificity of differential gene expression analysis in real data for derfinder, ShortStack, srnadiff, featureCounts and HTSeq.

Tool Type	Tool	Sensitivity	Specificity
Annotation-based	featureCounts	0.80	0.95
	HTSeq	0.73	0.96
Annotation-agnostic	derfinder	0.69	0.96
	snradiff	0.69	0.96
	ShortStack	0.67	0.94

Standardized differential expression analysis was performed with the edgeR package using data from GSE67004. Sensitivity and specificity were calculated based on the true positives and true negatives from differential gene expression using the authors parameters.

Table S4: Spearman correlation of feature quantification between annotation-based tools and annotation-agnostic methods with real data.

	HTSeq	derfinder	srnadiff	ShortStack
featureCounts	**0.993 ± 0.001**	**0.842 ± 0.019**	**0.650 ± 0.018**	**0.449 ± 0.029**
HTSeq		**0.852 ± 0.020**	**0.660 ± 0.018**	**0.451 ± 0.030**
derfinder			**0.649 ± 0.021**	**0.449 ± 0.036**
srnadiff				**0.385 ± 0.026**
ShortStack				

Known features were quantified using featureCounts, HTSeq, derfinder, srnadiff, and ShortStack. Read counts for features were correlated between methods on a per-sample basis. Values represent mean Spearman's rho ± standard deviation. Statistically significant correlations ($P<0.05$) are shown in bold.

Table S5: Comparison of direct mapping to miRbase and miRNA quantification using other expression quantification software.

**miRbase mapping**	**Normalized counts (All samples)**	**Effect-size (log2 fold-change)**	**FDR**
featureCounts	**0.948 ± 0.029**	**0.914**	**0.887**
HTSeq	**0.948 ± 0.029**	**0.915**	**0.887**
derfinder	**0.953 ± 0.025**	**0.912**	**0.883**
srnadiff	**0.943 ± 0.021**	**0.821**	**0.827**
ShortStack	**0.943 ± 0.020**	**0.922**	**0.869**

All statistics were calculated using the edgeR package. Statistically significant correlations to miRbase mapping are shown in bold. FC: Fold-change; FDR: False-Discovery Rate. Values are mean Spearman's rho ± standard deviation, where applicable.

Table S6: Spearman correlation of GO enrichment p-values between annotation-based tools and annotation-agnostic methods.

	HTSeq	derfinder	srnadiff	ShortStack
featureCounts	**0.99**	**0.78**	**0.68**	**0.61**
HTSeq		**0.77**	**0.68**	**0.61**
derfinder			**0.67**	**0.67**
srnadiff				**0.63**

Statistically significant correlation (p<0.05) are shown in bold. Values represent Spearman's rho.

Table S7: Spearman correlation of KEGG pathway enrichment p-values between annotation-based tools and annotation-agnostic methods.

	HTSeq	derfinder	srnadiff	ShortStack
featureCounts	**1.00**	**0.56**	**0.36**	**0.48**
HTSeq		**0.56**	**0.36**	**0.48**
derfinder			**0.32**	**0.37**
srnadiff				**0.66**

Statistically significant correlation (p<0.05) are shown in bold. Values represent Spearman's rho.

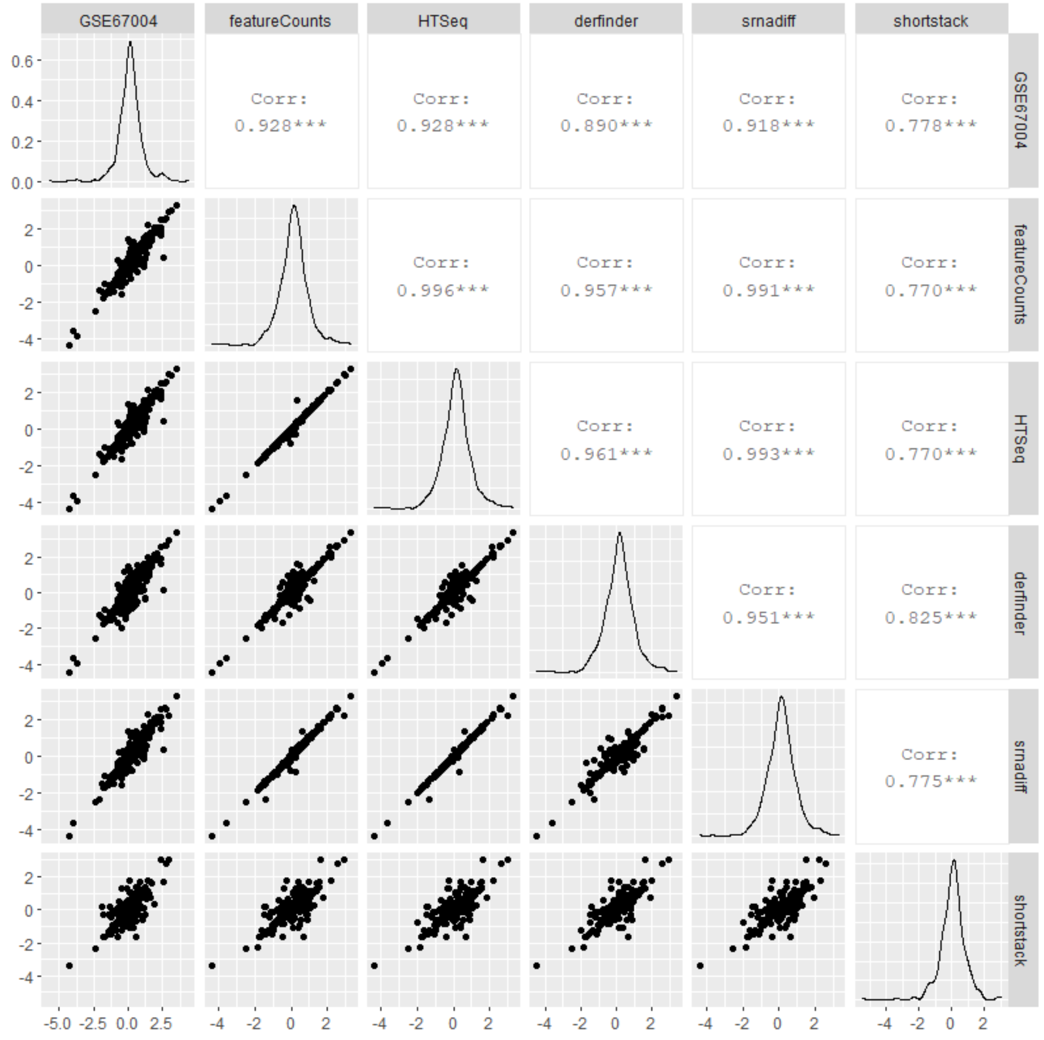

Figure S1: Annotation-agnostic tools and classical feature-based tools yield similar differential expression results. Log2 fold-change values calculated between EVs from KRAS-mutant cells and those from wild-type cells using data from GSE67004. Values represent spearman's correlation (rho) between log2 fold-change estimates between two tools. GSE67004 represents the truth set, calculated using the original author's method.

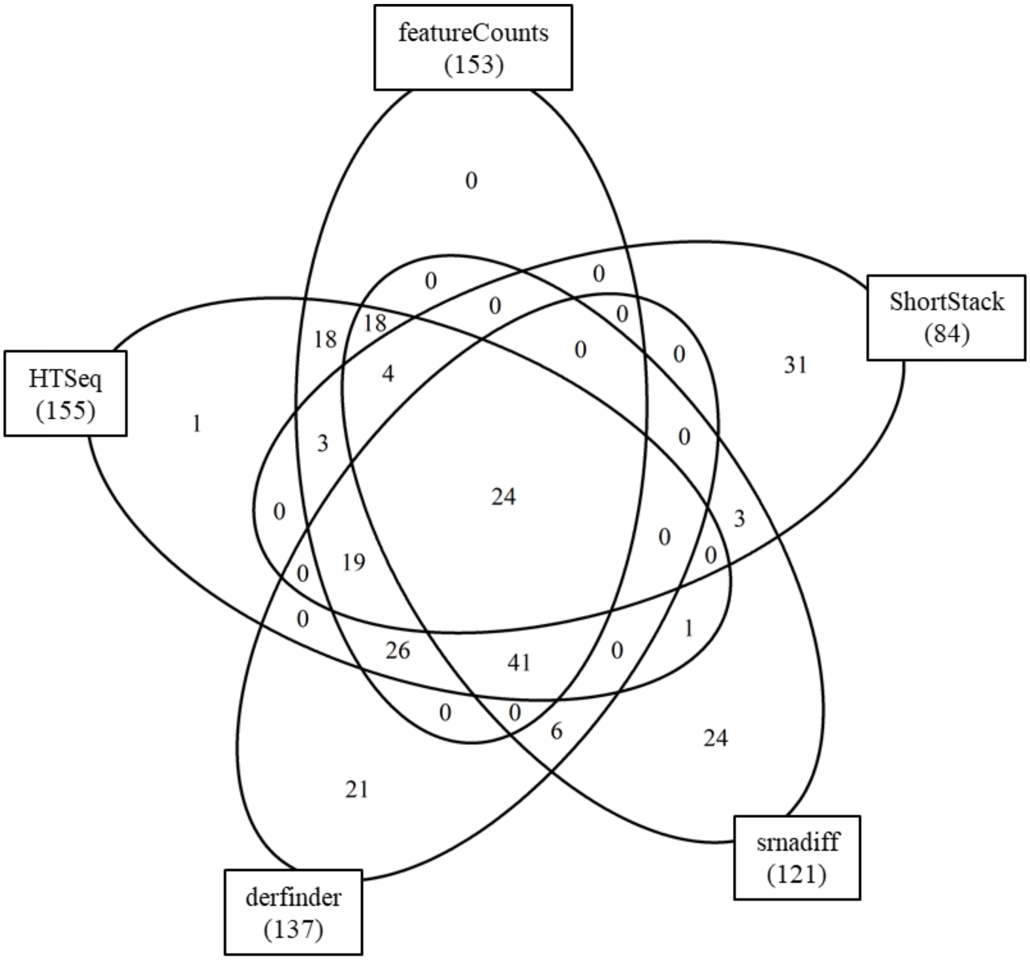

Figure S2: Overlap of standardized differential gene expression analysis results for all five tools. Count matrices were generated using derfinder, srnadiff, ShortStack, featureCounts, and HTSeq. Counts were then analyzed with the edgeR package for R using standardized parameters. Only statistically significant results were conserved.

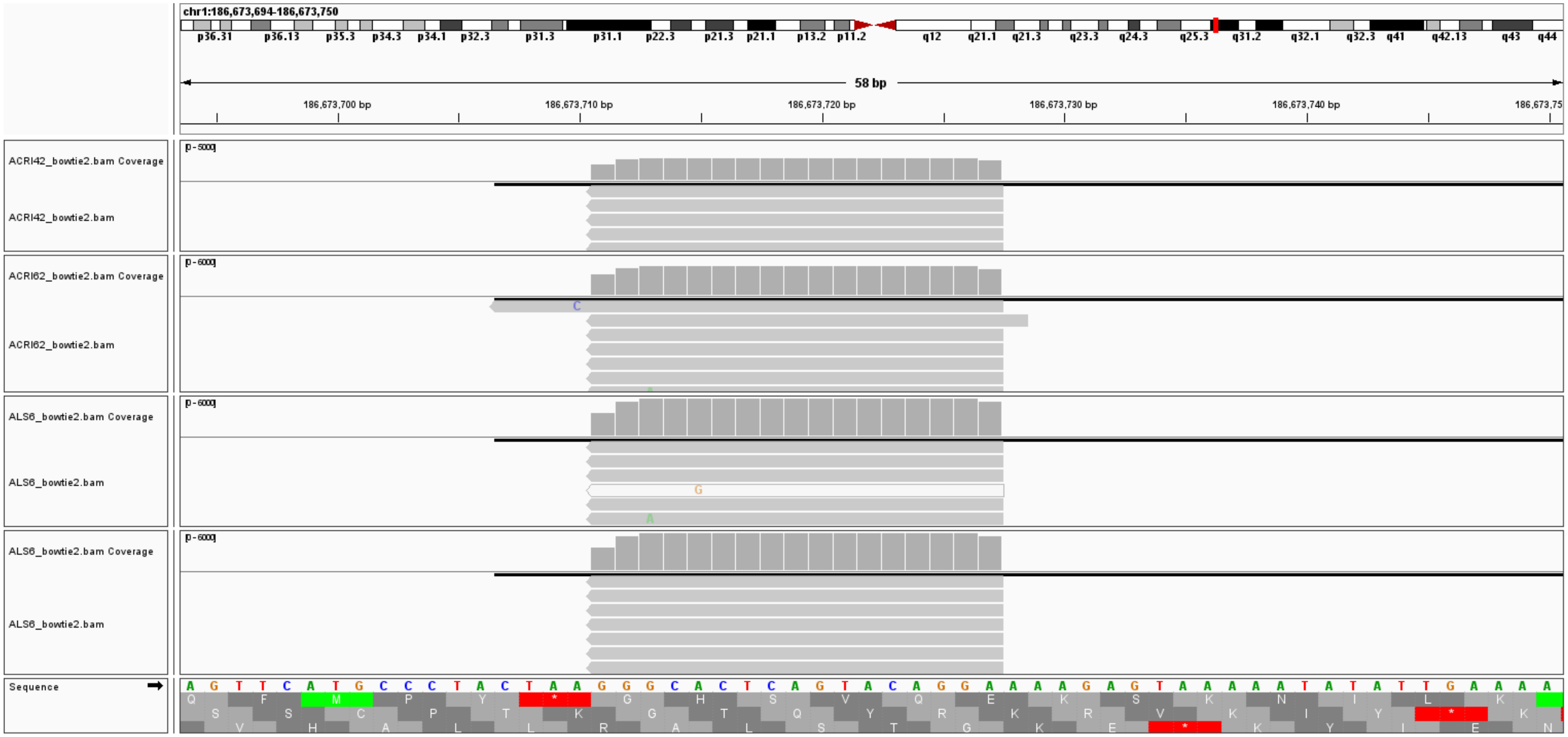

Figure S3: Validation target #1 is overexpressed in ALS.

An expressed RNA mapping to chr1:186673706-186673729 (hg19) was chosen for digital droplet PCR (ddPCR) validation as an EV-based expression biomarker for ALS. The total expressed region was 21 bases wide with a core sequence of 17 bases with high coverage.

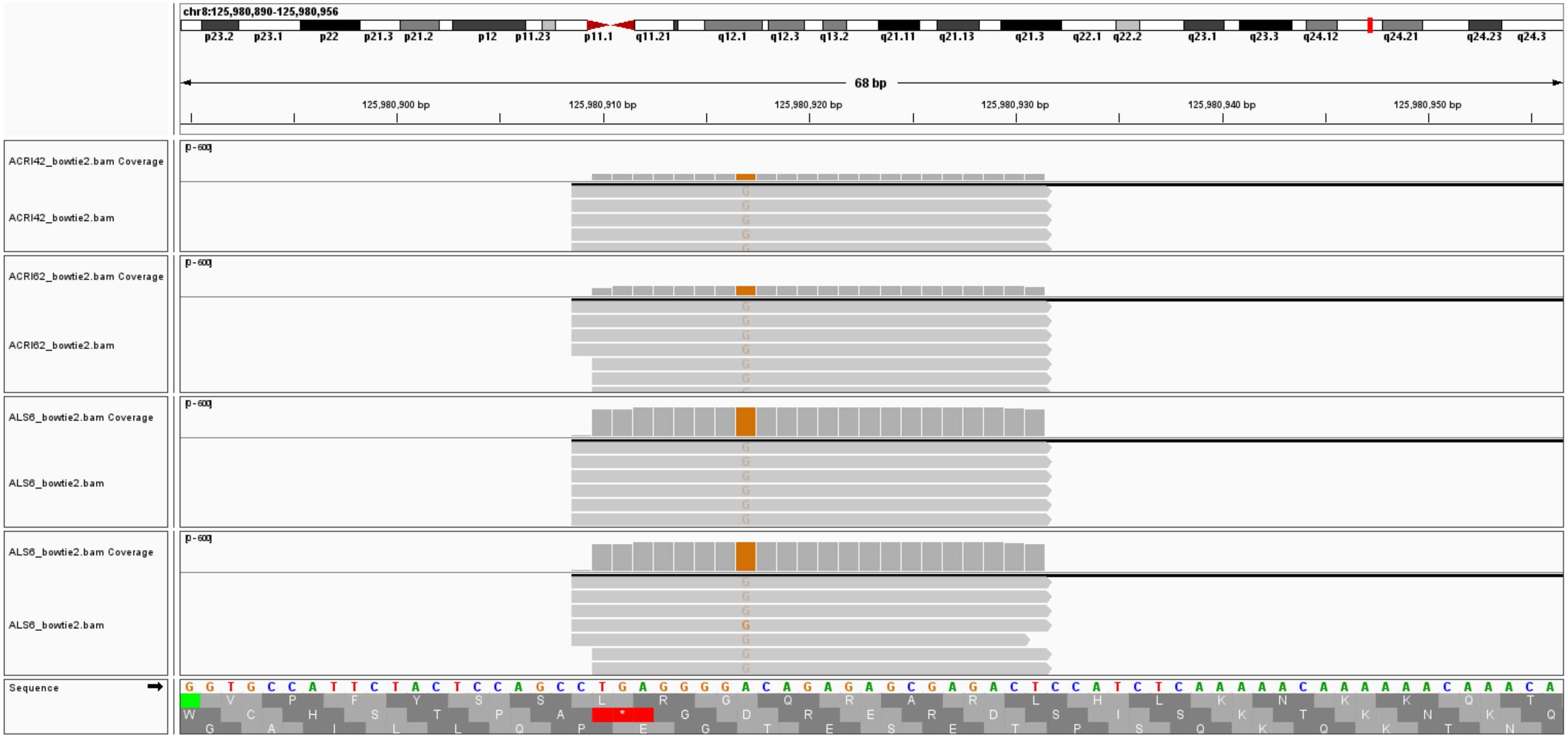

Figure S4: Validation target #1 is overexpressed in ALS.
An expressed RNA mapping to chr8:125980908-125980931 (hg19) was chosen for digital droplet PCR (ddPCR) validation as an EV-based expression biomarker for ALS. The total expressed region was 23 bases wide with a core sequence of 20 bases with high coverage. Most reads also reported an A > G mismatch with low quality, however this is likely a base quality error due to the low confidence call at this position.
